# Employees’ experiences of chronic pain in the workplace

**DOI:** 10.1093/occmed/kqaf052

**Published:** 2025-06-30

**Authors:** H Blake, M Giannoulatou, W J Chaplin

**Affiliations:** School of Health Sciences, University of Nottingham, Nottingham, UK; NIHR Nottingham Biomedical Research Centre, Nottingham, UK; School of Medicine, University of Nottingham, Nottingham, UK; School of Health Sciences, University of Nottingham, Nottingham, UK; NIHR Nottingham Biomedical Research Centre, Nottingham, UK

## Abstract

**Background:**

Between one-third and one-half of the UK population is affected by chronic pain. Effectively supporting people with chronic pain at work requires an understanding of employees’ experiences and expressed support needs.

**Aims:**

To understand how chronic pain affects people in their place of work, their reported support needs with relation to self-managing their chronic pain at work, and views towards the support provided by their employers.

**Methods:**

Qualitative study involving semi-structured interviews conducted with working-age adults who experience chronic pain and are employed in organizations in England. Data were analysed thematically and inductively.

**Results:**

Thirteen employees were interviewed (12 female, 1 male; aged 19–58 years). Four themes and 12 sub-themes were identified: (i) flexibility (hybrid working, working hours, manager support), (ii) inadequate support services (underdeveloped policies, poorly trained staff, inaccessibility), (iii) working conditions (equipment and adjustments, nature of job, being overworked), and (iv) perception of pain (stigma and discrimination, awareness and knowledge, support networks).

**Conclusions:**

This study provides insights into a range of factors that are described as helping or hindering the self-management of chronic pain at work. While support needs vary, inequities in workplace provisions and support are described. Occupational health and well-being services are described as not uniformly accessible, and workplace policies relating to chronic conditions or disability as vague. Line managers are described as playing a critical role in employee experiences, but are often perceived to lack the knowledge and training to address employees’ support needs.

## Teaser

This qualitative study focused on how people experience chronic pain at work. Employees with chronic pain placed high value on flexible and hybrid working, and employee support networks. Inequity in access to protective workplace policies, occupational health and well-being services was described. Participants referred to a ‘line manager lottery’ in which managers could either facilitate or hinder access to supportive intervention depending on their understanding and knowledge of the impact of chronic conditions on work.

## INTRODUCTION

Chronic pain is defined as pain that persists or recurs for more than 3 months [1]. It can be the sole or leading complaint of chronic pain syndromes, a condition in its own right (chronic primary pain) or secondary to an underlying health condition [1,2]. Affecting around 28 million adults in the UK [3], around 8 million adults experience chronic pain that is moderately to severely disabling [4]. An incidence growth of 32% is predicted by 2040, driven by an ageing population and the increase in the number of people living with multiple health conditions [5]. This will increase pressure on primary healthcare services, where many chronic pain conditions are managed [5]. Chronic pain impacts negatively on physical and mental health and leads to poorer quality of life compared to those with other long-term conditions and the general population [6].

In the context of work, chronic pain can lead to unemployment, loss of earnings, and impact on return-to-work for those who are absent [7]. Data from the Office for National Statistics shows that 9.3 million working-age people were economically inactive in 2024 [8]. In the UK, chronic pain can be classified as a disability under the Equality Act 2010. Disabled people are more likely to be economically inactive, with the majority citing long-term sickness as their main reason for being inactive [9]. Employees with pain symptoms have significantly higher work absenteeism [10] and lower work productivity [11] than those without pain, incurring significant economic impact for employers.

Supporting people to self-manage chronic pain at work may, therefore, benefit individual health, well-being and work participation, as well as organizational outcomes. However, this relies on understanding employees’ experiences and support needs.

The workplace experiences of employees with chronic pain have previously been studied to a limited extent [12,13]. In a diary study, Fragoso and McGonagle [12] examined 86 employees’ experiences of pain, how it interfered with daily activities at work and the link with psychological distress. They did not examine employees’ experiences of employer support or their specific support needs. In a survey study [13] with 274 employees with chronic pain, employees raised concerns and challenges relating to managing chronic pain at work and believed that awareness-raising efforts were needed. Organizational policies and practices were described as variable, and it was advocated that line managers should be trained in how chronic pain impacts people at work and how to provide reasonable adjustments. Recent changes in the labour market [8,9] and ways of working since the coronavirus disease 2019 (COVID-19) pandemic [14] mean an updated exploration of experiences, support needs, and employer provisions is warranted to inform future supportive interventions. Qualitative research can draw new and more in-depth insights into employee experiences and support needs, but is lacking in this field.

Therefore, the aim of the study is to qualitatively explore how chronic pain affects people in their place of work, their reported support needs with relation to self-managing chronic pain at work and their views towards the support provided by their employers. The research question is: how is chronic pain experienced in the workplace?

## METHODS

This was a qualitative study using semi-structured interviews. The study was approved by the University of Nottingham MSc Health Psychology Ethics Review panel on the 2 May 2024 (REC reference: MEDS4008-24-02). The study design adhered to the consolidated criteria for reporting qualitative studies (COREQ) guidelines [15] (File S1, available as Supplementary data at *Occupational Medicine* online). Participants were a convenience sample of individuals aged 18 years or over, who self-reported chronic pain, and were employed in England, recruited using a study invitation circulated via disability networks, pain charities and social media postings. We used an interview topic guide (File S2, available as Supplementary data at *Occupational Medicine* online), which was pilot tested. Interview data were collected over 6 weeks between May and July 2024, using Microsoft Teams. Interviews were audio-recorded with consent and fully transcribed. Written informed consent and demographic data were obtained via Jisc Online Surveys accessed through an email link or a QR code, with additional verbal consent audio-recorded prior to the interview. Field notes were taken. Interviews were conducted by one researcher (M.G.). Initial analysis was undertaken by one researcher (M.G.) who undertook coding and developed the themes. Themes were then refined and discussed with a second researcher (H.B.). All researchers had a background in health psychology and an interest in chronic pain and work. Participants were offered the opportunity to enter a prize draw for a £50 high street shopping voucher. Interview length varied from 11 to 42 minutes (mean: 23.07 minutes). Recruitment continued until thematic saturation was reached [16]. Data were analysed using a six, non-linear step process of thematic analysis involving: familiarisation with data, generating initial codes, searching for themes, reviewing themes, defining and naming themes, and finally producing the report, following an inductive approach [17].

## RESULTS

Twenty-three people expressed initial interest in taking part, 13 participants completed an interview. Those who expressed interest but did not take part were too busy, could not find a convenient time for interview or did not respond to the researcher’s email. Participants’ characteristics are shown in Tables 1 and 2.

**Table 1.** Participants’ gender, age and ethnicity

Participant ID	Gender	Age (years)	Ethnicity
IDP1	Female	58	White
IDP2	Non-binary	19	White
IDP3	Female	45	Asian
IDP4	Female	48	Asian
IDP5	Male	30	White
IDP6	Female	52	White
IDP7	Female	31	White
IDP8	Non-binary	26	White
IDP9	Female	39	White
IDP10	Female	56	White
IDP11	Female	26	White
IDP12 IDP13	Female Female	23 33	White White

**Table 2.** Participants’ type and duration of pain, company size, employment status and workplace.

Participant ID	Type of pain	Duration of pain	Company size	Employment status	Workplace
IDP1	Arthritis and migraines	30 years	Large	Full-time	Hybrid
IDP2	Connective tissue, joints/bones	19 years	Large	Full-time	Hybrid
IDP3	Nerve	10 years	Large	Full-time	Hybrid
IDP4	Joint, muscle, bone	Lifelong	Large	Full-time	Hybrid
IDP5	Lower back	2 years	Large	Full-time	Hybrid
IDP6	Degenerated IV discs	18 years	Large	Part-time	Place of work
IDP7	Period pain, migraines	18 years	Large	Full-time	Place of work
IDP8	Neck, back, migraines	10 years	Large	Full-time	Hybrid
IDP9	Right hip joint	20+ years	Large	Part-time	Hybrid
IDP10	Joints, muscles	4 years	Large	Part-time	Hybrid
IDP11	Back, neck	10 years	Large	Full-time	Hybrid
IDP12	Menstrual cramps	7 years	Large	Full-time	Hybrid
IDP13	Endometriosis, fibromyalgia	11 years	Micro	Part-time	At home

**Figure 1.**
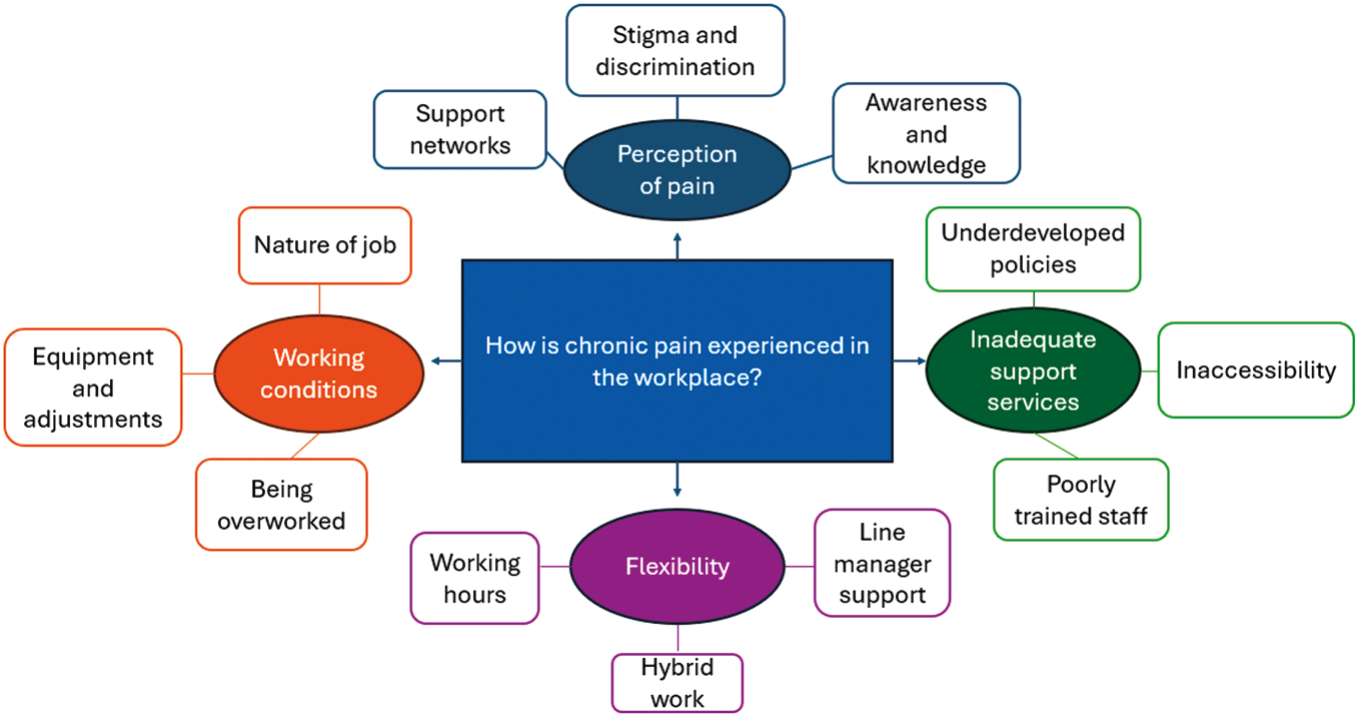
Thematic map illustrating the final themes and sub-themes

Analysis of interviews generated four final themes including flexibility, inadequate support services, working conditions and perception of pain, and their 12 respective sub-themes (Figure 1).

Most participants benefited from flexible working hours, which provided periods of recovery from pain symptoms during the working day.

If I ever feel unwell in the afternoon, for example, I’m allowed to actually catch up … a bit at a time on other days … just to … make up for it. (IDP12)

I tend to split my work up, I’m quite fortunate that I can work fairly flexibly with my hours, so instead of doing one 9-5 stint, I’ll do two or three hours, I’ll go off and do something else, try and get rid of the pain. (IDP8)

Some participants were unable to work flexible hours due to the specific requirements of their job role, which created challenges for them in managing fluctuating symptoms.

Most participants engaged in hybrid work (partly on work premises, partly at home), which was highly valued, provided a sense of autonomy and control over their working environment, and facilitated self-management of pain and fatigue. However, not all employers sanctioned hybrid work patterns.

I can decide to stay at home or to go to office … going to the office is not compulsory. (IDP5)

I thought it might help me if I worked only one day in the office and three days at home … it wasn’t allowed. (IDP9)

Hybrid and flexible working were more likely to be in place when employees had a supportive line manager with knowledge about chronic pain and the processes for making reasonable adjustments. Line managers’ awareness and understanding were perceived to vary significantly, which put access to support down to luck.

we call it the ‘line manager lottery’ in terms of whether your line manager is supportive or knowledgeable. (IDP8)

Participants perceived that line managers who had experienced a chronic condition themselves had greater understanding of the impact on work and were more willing to facilitate access to support than those who had no personal experience.

I’ve got a very lenient manager because she’s also got chronic health issues. (IDP11)

Views towards occupational health (OH) services were highly variable. Services were provided in-house or by external companies, only for those working in the large organizations. Broadly, in-house services were valued, particularly when line managers were supportive of the OH advisor’s recommendations. However, only two employees referred to having access to in-house services. External services, accessible to the remaining interviewees, were perceived to be less helpful, as participants felt that the advice was not relevant to them, or their struggles with pain at work or within their job role were not understood. They perceived that the external OH staff were often poorly trained.

OH tends to be hit and miss, and the knowledge of what role OH plays is very hit and miss. (IDP8)

it was a box ticking exercise all round. It felt like they weren’t actually of any help. (IDP9)

Some participants were unaware of any policies to protect people with long-term conditions (relating to disability and sickness absence). Those who had viewed policies generally found them vague and unhelpful.

… they’re just surface level policies. They’re not actually doing the groundwork of actually … helping people with physical disabilities. (IDP11)

very light on details…most of it comes down to the manager's discretion … (IDP9)

Participants experienced barriers to accessing supportive services. Long waiting times for OH appointments were a particular problem. Well-being activities were offered in some organizations but were not always accessible to people with chronic pain, if events involved sitting for long periods, or travelling to attend them.

they made me sit for two hours without moving … I was in so much pain. (IDP1)

Some participants highlighted that their employer focused less on workforce well-being and more on customer well-being. For example, participants from the education sector reported that well-being-focused events and supportive services were accessible to students, but inaccessible to staff.

Many participants had received equipment as an adjustment to help them to manage their pain at work, including chairs, standing desks and footrests.

sitting standing desks … so I can do a bit of work standing up, it’s great for my back … I have a little footrest … I have a good comfy chair with a back support bit built in. (IDP8)

Some organizations allowed employees to bring items in from home to facilitate their comfort and relieve pain, such as hot water bottles. Other participants were unable to obtain the equipment they needed, due to a lack of budget or poor support from line managers. An inability to adjust the working environment to minimize their pain increased participants’ desire to work from home as a pain management strategy.

Those with desk-based jobs generally found it easier to self-manage their pain as adjustments to working hours and the work environment were more feasible. Those with physically demanding jobs, involving regular lifting or standing, found pain management in the workplace too challenging. For some, this had led to a permanent reduction in working hours and therefore loss of pay, or a change of career.

I’ve … cut my hours down … I was on my feet all the time, I couldn’t manage that … I changed to teaching … a good variety of sitting and standing, which makes it easier for me. (IDP6)

High workloads were commonly experienced, described as ‘becoming normalised’, and were a key factor exacerbating pain, impacting on physical and mental well-being, and influencing pain self-management. Supportive interventions for health and well-being were then perceived to be ‘tick-box’ exercises that failed to fix the fundamental issue of excessive workloads. This generated frustration and anger towards employers.

they can’t expect to just keep throwing well-being tools and well-being event setters and that will fix the problem … if we can’t fix workload, there needs to be … robust support for staff. (IDP8)

Many participants referred to a lack of awareness and knowledge within their organization relating to physical health conditions, particularly related to the diversity in individual experiences of pain, and the day-to-day fluctuations in pain levels. Those with non-visible pain conditions felt unseen in their workplace.

Today it’s my right shoulder and my left hip that hurts me, but tomorrow it could be somewhere else … depending on what joint it is, actually affects the way I’m working. (IDP10)

Some participants shared experiences of stigma and discrimination in the workplace associated with their long-term condition. For some, perceived stigma led to fear of losing their job or prevented them from disclosing a disability at work or accessing support.

… because of my pain levels … because I’m disabled, I was seen as a risk to others. (IDP1)

You do worry … that there’s any kind of negative impact in sharing that sort of information … you … keep it to yourself a little bit, until such a time that you might need … help. (IDP7)

However, other employees had not experienced discrimination and reported working in inclusive environments.

Participants disclosed that having access to a support network (e.g. Disability Staff Network) within their organization or sector, with people facing similar struggles, can help them feel more optimistic in managing their condition. These were opt-in groups where employees who self-identified as having a disability could receive regular notifications and updates relating to relevant policies and practices within their organization and attend meetings where issues relating to work and disability could be confidentially discussed with peers.

Quite empowering really … you feel able to talk about it, you don’t feel the need to … hide it in case it’s perceived as a weakness or … a negative thing. (IDP13)

Although not all participants were aware of, or had accessed support networks, those who had alluded to psychologically safe environments where they could speak openly about their condition and find shared solutions to self-management.

I think there’s an understanding that it’s not what disabilities you have, but what abilities you have and just having … colleagues at all levels and in all teams who understand that. (IDP4)

## DISCUSSION

This qualitative interview study identified four key themes associated with employees’ experiences of chronic pain at work: flexibility, inadequate support services, working conditions and perceptions of pain. These themes highlighted that although experiences of working with chronic pain were individual, diverse and fluctuating, there were common factors across the sample. Overall, self-management of chronic pain was facilitated by flexible and hybrid working patterns, access to reasonable adjustments through managers or in-house OH services, manageable workloads and employee support networks for staff with disabilities, which created a psychologically safe environment to discuss work impacts and solutions. Key barriers to self-management of chronic pain were workplace stigma and discrimination, lack of awareness and knowledge about chronic conditions and processes for support, unmanageable workloads, and inadequate support founded in vague policies, lack of access to OH and well-being services, and advice perceived to be poor or irrelevant; largely provided by external OH services. The role of line managers is seen to be critical, acting as either a barrier or enabler of disability disclosure, access to support and, ultimately, chronic pain self-management in the workplace.

In recent years, there has been a dramatic shift in ways of working, and 40% of employers have observed an increase in formal requests for flexible working following the COVID-19 pandemic [18]. As found elsewhere, flexibility over location and working hours increases autonomy and control for employees, allowing those with disabilities and chronic health conditions to better manage their health and well-being and be more productive at work [19,20]. The 2021 Chartered Management Institute (CMI) survey recommended that inclusive flexible working should be the default position for employers [19,20]. However, while 85% of organizations in the CMI survey offered hybrid working, only 45% had a policy relating to it, which concurs with our participants’ reports that workplace policies relating to inclusive working were not always clear, or in place.

The critical role of line managers in supporting the health and well-being of the workforce is outlined in previous research [12,21] and in national guidelines and quality standards, which advocate the need for line manager training to equip them with the knowledge and skills to provide support [22–24]. Our findings show that support from line managers is not uniformly described, yet line managers play a critical role in facilitating flexible working patterns and other reasonable adjustments that empower people to self-manage their chronic pain. Inadequate support was often flagged by those with non-visible pain conditions. There is a continued need for line manager training to increase awareness of chronic pain, its impacts on work participation and routes to employee support.

This qualitative study is limited by its small sample size and convenience-based sampling method. Most of our participants were employed in large organizations, so our findings will not reflect the views of those working in small- to medium-sized enterprises (SMEs). SMEs report lower access to OH services [25] and a lack of resources and knowledge as barriers to implementing health and well-being services [26]. Most interviewees were women, which could reflect the gender imbalance in chronic pain experience and reporting [27] or the challenges of recruiting men to health research studies [28]. Although small, our sample size was sufficient to capture a comprehensive range of issues in our data (‘code saturation’). While data saturation is a core guiding principle to determine sample size in qualitative research, the diversity of our population in terms of chronic pain conditions and employment settings means that a larger sample size may be needed to achieve both ‘code’ and ‘meaning’ saturation, that is, a richly textured understanding of those issues [16].

Despite limitations of study design, we provide new insights into the experiences of people with chronic pain at work and their support needs, against a backdrop of limited research literature in this area. Further research is needed to examine workplace policies to support people at work with chronic pain, how and where they are implemented, and the extent to which they fulfil the needs of employees with such long-term conditions. We advocate that employers create psychologically safe work environments where stigma and discrimination are not tolerated, and employees feel comfortable to disclose their condition and liaise with line managers over workloads, reasonable adjustments and support. The knowledge and support of line managers were described as variable, which aligns with research conducted prior to the COVID-19 pandemic [12]. This persistent finding suggests that line manager training to increase knowledge of chronic pain and disabilities more broadly, including non-visible conditions, may prove supportive. This is in keeping with the position of professional bodies in relation to support for chronic conditions (e.g. The Chartered Institute of Personnel and Development) [29], and with prior research on chronic pain [12] and other health conditions (e.g. mental health [30]) in the workplace.

**Key learning points**

**What is already known about this subject:**

- A high proportion of the UK population is affected by chronic pain.
- Many people who live with chronic pain experience challenges in self-managing their condition at work.
- Effectively supporting people to self-manage chronic pain at work requires a current understanding of employees’ experiences and support needs.

**What this study adds:**

- Employees with chronic pain placed high value on flexible and hybrid working and employee support networks.
- There was inequity in access to protective workplace policies, occupational health and well-being services.
- Line managers could either facilitate or hinder access to supportive intervention depending on their understanding and knowledge of the impact of chronic conditions on work.

**What impact this may have on practice or policy:**

- Efforts may be needed to increase equity in access to occupational health services or support for working adults with chronic pain.
- Research is needed to further examine workplace policies to support people at work with chronic pain, how and where they are implemented, and the extent to which they fulfil the needs of employees with long-term conditions.
- Line manager training may help to increase knowledge of the impacts of chronic pain at work and ensure managers can implement reasonable adjustments that will enhance work capacity in employees with chronic pain.

## Supplementary Material

kqaf052_suppl_Supplementary_File_S1

kqaf052_suppl_Supplementary_File_S2

## References

[1] Treede RD , Rief W, Barke A et al Chronic pain as a symptom or a disease: the IASP Classification of Chronic Pain for the International Classification of Diseases (ICD-11). Pain 2019;160:19–27. doi: https://doi.org/10.1097/j.pain.0000000000001384 30586067

[2] *ICD-11 International Classification of Diseases 2023*. https://www.who.int/standards/classifications/classification-of-diseases

[3] Fayaz A , Croft P, Langford RM, Donaldson LJ, Jones GT. Prevalence of chronic pain in the UK: a systematic review and meta-analysis of population studies. BMJ Open 2016;6:e010364. doi: https://doi.org/10.1136/bmjopen-2015-010364 PMC4932255 27324708

[4] Von Korff M , Ormel J, Keefe FJ, Dworkin SF. Grading the severity of chronic pain. Pain 1992;50:133–149. 1408309 10.1016/0304-3959(92)90154-4

[5] The Health Foundation. *Health in 2040: Projection Patterns of Illness in England (2023)*. https://www.health.org.uk/reports-and-analysis/reports/health-in-2040-projected-patterns-of-illness-in-england (Updated September 2023).

[6] Hadi MA , McHugh GA, Closs SJ. Impact of chronic pain on patients’ quality of life: a comparative mixed-methods study. J Patient Exp. 2019;6:133–141. doi: https://doi.org/10.1177/2374373518786013 31218259 PMC6558939

[7] Dobson KG , Mustard C, Carnide N, Furlan A, Smith PM. Impact of persistent pain symptoms on work absence, health status and employment 18 months following disabling work-related injury or illness. Occup Environ Med 2022;79:697–705. 35902222 10.1136/oemed-2022-108383 PMC9484373

[8] Office for National Statistics. Economic Inactivity. Economic Inactivity. Data and analysis from Census, 2021. https://www.ons.gov.uk/employmentandlabourmarket/peoplenotinwork/economicinactivity (12 February 2025, date last accessed).

[9] Department for Work and Pensions. *Official Statistics: Employment of Disabled People 2023*. https://www.gov.uk/government/statistics/the-employment-of-disabled-people-2023/employment-of-disabled-people-2023#:~:text=Disabled%20people%20were%20more%20likely%20to%20be%20economically%20inactive%20and,in%20the%20last%20two%20years

[10] Adams G , Salomons TV. Attending work with chronic pain is associated with higher levels of psychosocial stress. Can J Pain 2021;5:107–116. doi: https://doi.org/10.1080/24740527 34189394 PMC8210861

[11] Kawai K , Kawai AT, Wollan P, Yawn BP. Adverse impacts of chronic pain on health-related quality of life, work productivity, depression and anxiety in a community-based study. Fam Pract 2017;34:656–661. doi: https://doi.org/10.1093/fampra/cmx034 28444208 PMC6260800

[12] Blake H , Somerset S, Greaves S. The Pain at work toolkit for employees with chronic or persistent pain: a collaborative-participatory study. Healthcare 2022;10:56. doi: https://doi.org/10.3390/healthcare10010056 PMC8775489 35052220

[13] Fragoso ZL , McGonagle AK. Chronic pain in the workplace: a diary study of pain interference at work and worker strain. Stress Health 2018;34:416–424. doi: https://doi.org/10.1002/smi.2801 29484812

[14] McKinsey Global Institute. The future of work after COVID-19, February 18, 2021, Report. https://www.mckinsey.com/featured-insights/future-of-work/the-future-of-work-after-covid-19

[15] Tong A , Sainsbury P, Craig J. Consolidated criteria for reporting qualitative research (COREQ): A 32-item checklist for interviews and focus groups. Int J Qual Health Care 2007;19:349–357. 17872937 10.1093/intqhc/mzm042

[16] Hennink MM , Kaiser BN, Marconi VC. Code Saturation versus meaning saturation: how many interviews are enough? Qual Health Res 2017;27:591–608. 27670770 10.1177/1049732316665344 PMC9359070

[17] Braun V , Clarke V. Using thematic analysis in psychology. Qual Res Psychol 2006;3:77–101.

[18] CIPD. *Flexible and Hybrid Working Practices in 2023: Employer and Employee Perspectives*. https://www.cipd.org/globalassets/media/knowledge/knowledge-hub/reports/2023-pdfs/2023-flexible-hybrid-working-practices-report-8392.pdf (May 2023).

[19] Taylor H , Florisson R, Hooper D. *Making Hybrid Inclusive: Key Priorities for Policymakers*. Chartered Management Institute. https://www.managers.org.uk/wp-content/uploads/2021/10/WF-CMI-2021-Policy-brief-making-hybrid-inclusive.pdf (October 2021).

[20] Taylor, H, Florisson, R, Wilkes, M and Holland, P. (2022) The Changing Workplace: Enabling Disability-Inclusive Hybrid Working. Work Foundation, Lancaster University.

[21] Gulseren D , Kelloway EK. Working through the pain: the chronic pain experience of full-time employees. Occup Health Sci 2021;5:69–93. doi: https://doi.org/10.1007/s41542-020-00078-x

[22] CIPD. Health and Wellbeing at Work. London: Chartered Institute of Personnel and Development, 2023. https://www.cipd.org/globalassets/media/knowledge/knowledge-hub/reports/2023-pdfs/8436-health-and-wellbeing-report-2023.pdf

[23] Department for Work and Pensions. *Recruiting, Managing and Developing Disabled People: A Practical Guide for Managers*. https://www.gov.uk/government/publications/disability-confident-and-cipd-guide-for-line-managers-on-employing-people-with-a-disability-or-health-condition/guide-for-line-managers-recruiting-managing-and-developing-people-with-a-disability-or-health-condition#the-role-of-the-manager (Updated 24 May 2024).

[24] National Institute for Health and Care Excellence. *Healthy Workplaces: Improving Employee Mental and Physical Health and Wellbeing. Quality Standard [QS147] Published*. https://www.nice.org.uk/guidance/qs147/chapter/quality-statement-2-role-of-line-managers#:~:text=Organisations%20also%20ensure%20that%20line,job%20descriptions%20and%20performance%20reviews (03 March 2017).

[25] Department for Work and Pensions. *Employee Research Phase 1: Sickness Absence, Reasonable Adjustments and Occupational Health*. DWP report no. 1021. https://www.gov.uk/government/publications/employee-research-phase-1-and-2/employee-research-phase-1-sickness-absence-reasonable-adjustments-and-occupational-health#:~:text=Employee%20access%20to%20OH%20varies,10%25) (Updated 15 March 2023).

[26] Burge P , Lu H, Smith P, Koch N. *Incentivising SME Uptake of Health and Wellbeing Support Schemes*. Research Report no. 1024. Department of Work and Pensions. https://assets.publishing.service.gov.uk/media/640f0d67d3bf7f02ff3f5744/incentivising-SME-uptake-of-health-and-wellbeing-schemes-report.pdf (March 2023).

[27] Osborne NR , Davis KD. Sex and gender differences in pain. Int Rev Neurobiol 2022;164:277–307. doi: https://doi.org/10.1016/bs.irn.2022.06.013 36038207

[28] Borg DJ , Haritopoulou-Sinanidou M, Gabrovska P et al Barriers and facilitators for recruiting and retaining male participants into longitudinal health research: a systematic review. BMC Med Res Methodol 2024;24:46. doi: https://doi.org/10.1186/s12874-024-02163-z 38389065 PMC10882922

[29] The Chartered Institute for Personnel and Development. 2025. *Long-Term Health Conditions: How People Professionals Can Support Employees*. https://www.cipd.org/en/knowledge/guides/support-long-term-health-conditions/.

[30] Blake H , Hassard J, Dulal-Arthur T et al Typology of employers offering line manager training for mental health. Occup Med (Lond) 2024;74:242–250. doi: https://doi.org/10.1093/occmed/kqae025 38722211 PMC11080657

