# Supplementary material for: Employees’ experiences of chronic pain in the workplace
Source: Occup Med (Lond). 2025 Jun 30;75(5):250–5. doi: 10.1093/occmed/kqaf052 (PMC12370396; doi:10.1093/occmed/kqaf052)
Supplement: kqaf052_suppl_Supplementary_File_S1 [file kqaf052_suppl_supplementary_file_s1.docx]

**Supplementary file S1.** Consolidated criteria for reporting qualitative studies (COREQ): 32-item checklist

| **Topic and Item No.** | **Guide Questions/Description** | **Response** |
| --- | --- | --- |
| **Domain 1: Research team and reflexivity** | | |
| *Personal Characteristics* | | |
| 1. Interviewer/facilitator | Which author/s conducted the interview or focus group? | Maria Giannoulatou |
| 2. Credentials | What were the researcher’s credentials? E.g. PhD, MD | MSc |
| 3. Occupation | What was their occupation at the time of the study? | Early career researcher |
| 4. Gender | Was the researcher male or female? | Female |
| 5. Experience and training | What experience or training did the researcher have? | Prior experience in qualitative interviews and analysis  Trained in research methods and good clinical practice (GCP) |
| *Relationship with participants* | | |
| 6. Relationship established | Was a relationship established prior to study commencement? | Researchers met the participants during recruitment |
| 7. Participant knowledge of the interviewer | What did the participants know about the researcher? e.g. personal goals, reasons for doing the research | Participants knew that the interviewer was a university researcher and registered student. |
| 8. Interviewer characteristics | What characteristics were reported about the interviewer/facilitator? e.g. Bias, assumptions, reasons and interests in the research topic | The interviewer was a White female, in the 21-30 age range. Participants knew that MG was conducting Masters level study and had an interest in the experiences of people with chronic conditions in the workplace. |
| **Domain 2: Study design** |  |  |
| *Theoretical framework* | | |
| 9. Methodological orientation and theory | What methodological orientation was stated to underpin the study? e.g. grounded theory, discourse analysis, ethnography, phenomenology, content analysis | Inductive thematic analysis |
| Participant selection | | |
| 10. Sampling | How were participants selected? e.g. purposive, convenience, consecutive, snowball | Convenience sample |
| 11. Method of approach | How were participants approached? e.g. face-to-face, telephone, mail, email | Participants were approached and recruited via an advertisement circulated on social media, and via distribution lists of charities and professional networks |
| 12. Sample size | How many participants were in the study? | 13 |
| 13. Non-participation | How many people refused to participate or dropped out? Reasons? | No participants actively withdrew from the study. 10 further participants expressed an interest but then were not available for interview during the recruitment window. |
| *Setting* | | |
| 14. Setting of data collection | Where was the data collected? e.g. home, clinic, workplace | Data were collected online (video-conferencing platform) |
| 15. Presence of non-participants | Was anyone else present besides the participants and researchers? | No |
| 16. Description of sample | What are the important characteristics of the sample? e.g. demographic data, date | Working adults self-identifying as having chronic pain  12 female, 1 male; aged 19-58 years |
| *Data collection* | | |
| 17. Interview guide | Were questions, prompts, guides provided by the authors? Was it pilot tested? | Yes. The questioning guide was pilot tested with individuals who were not participants in this study. |
| 18. Repeat interviews | Were repeat interviews carried out? If yes, how many? | No repeat interviews. |
| 19. Audio/visual recording | Did the research use audio or visual recording to collect the data? | Interviews were audio-recorded using a video-conferencing platform. Interviewer kept camera on to assist with establishing rapport. |
| 20. Field notes | Were field notes made during and/or after the interview or focus group? | Yes. |
| 21. Duration | What was the duration of the interviews or focus group? | Interview length varied from 11 to 42 min (mean: 23.07 min) |
| 22. Data saturation | Was data saturation discussed? | Yes. |
| 23. Transcripts returned | Were transcripts returned to participants for comment and/or correction? | No due to time limitations. |
| **Domain 3: analysis and findings** |  |  |
| *Data analysis* | | |
| 24. Number of data coders | How many data coders coded the data? | One (with supervision from the other two authors). |
| 25. Description of the coding tree | Did authors provide a description of the coding tree? | No, however initial coding was informed by the interview guide, and coding was continuously  refined. |
| 26. Derivation of themes | Were themes identified in advance or derived from the data? | These were derived from the data. |
| 27. Software | What software, if applicable, was used to manage the data? | N’Vivo |
| 28. Participant checking | Did participants provide feedback on the findings? | Yes. |
| *Reporting* | | |
| 29. Quotations presented | Were participant quotations presented to illustrate the themes / findings? Was each quotation identified? e.g. participant number | Yes. |
| 30. Data and findings consistent | Was there consistency between the data presented and the findings? | Yes. |
| 31. Clarity of major themes | Were major themes clearly presented in the findings? | Yes. |
| 32. Clarity of minor themes | Is there a description of diverse cases or discussion of minor themes? | Yes. |
