# Supplementary material for: Employees’ experiences of chronic pain in the workplace
Source: Occup Med (Lond). 2025 Jun 30;75(5):250–5. doi: 10.1093/occmed/kqaf052 (PMC12370396; doi:10.1093/occmed/kqaf052)
Supplement: kqaf052_suppl_Supplementary_File_S2 [file kqaf052_suppl_supplementary_file_s2.docx]

**Supplementary file S2.** Interview Schedule

Demographic questions (to be asked before the recording begins)

1. What is your age?
2. What gender do you identify with?
3. What is your ethnicity?

Interview questions:

1. To understand how chronic pain affects people at work

- How does pain affect you while you are at work? (prompt?)
- Can you tell me a bit more about that?

1. To understand what employees with chronic pain need in their workplace to help manage their condition

- What activities does your job normally involve?
- Are there any particular things that help you to manage your pain while at work?

1. To understand what employers do to support their employees with chronic pain

- What sort of things does your employer have in place to support people who have chronic pain? (prompt?)
- Is there an occupational health service, or particular policies to protect people with long term conditions? Any well-being activities?
- Have you accessed any support from your employers?
- Ideally, what else would you like your employers to do to support you?
